# Unmasking the Hidden Complexity of Mandibular Premolars: A Case Series on Navigating Root Canal Variations in Endodontics

**DOI:** 10.7759/cureus.97537

**Published:** 2025-11-23

**Authors:** Prashant Bhasin, Lubna Ahmad, Sachin Chauhan, Palak Agrawal

**Affiliations:** 1 Conservative Dentistry and Endodontics, Sudha Rustagi College of Dental Sciences and Research, Faridabad, IND; 2 Dentistry, Hamdard Institute of Medical Sciences and Research and Hakeem Abdul Hameed Centenary (HAHC) Hospital, New Delhi, IND; 3 Endodontics, Dr Chauhan's Root Canal and Implant Centre, Faridabad, IND; 4 Dentistry, Mobident Dental Centre, New Delhi, IND

**Keywords:** bioceramics, case series, cone beam computed tomography, dental operating microscope, mandibular premolars, root canal variations

## Abstract

Mandibular premolars are often perceived as straightforward in endodontic therapy due to their typically single-rooted anatomy. However, an increasing body of evidence reveals significant variations in their root canal morphology, posing diagnostic and therapeutic challenges for clinicians. These anatomical anomalies, including multiple canals, bifurcations, and atypical curvatures, may lead to missed canals, inadequate debridement, and ultimately treatment failure if not properly identified and managed.

This case series showcases three cases exhibiting the types of root canal variations in mandibular premolars, emphasizing the importance of advanced diagnostic tools such as cone-beam computed tomography (CBCT), enhanced magnification, and illumination. The first case, a 44-year-old woman, exhibited two roots and three canals in both premolars, confirmed by CBCT imaging. Treatment involved rotary instrumentation, heated sodium hypochlorite irrigation, and obturation with bioceramic sealers under magnification. The second case featured a 33-year-old man with a previously initiated treatment in tooth #35. Enhanced visualization under the dental microscope revealed three separate canals, which were disinfected and obturated using a single-cone technique. The third case involved a 27-year-old woman whose premolar had a Type II configuration, with canals merging apically. Careful negotiation and sealing were performed under magnification. These cases highlight that combining CBCT, magnification, and contemporary obturation materials is essential for effectively managing complex premolar anatomies and achieving predictable outcomes.

The study also explores contemporary shaping, cleaning, and obturation techniques tailored to complex canal systems. By integrating anatomical knowledge with evolving clinical strategies, endodontists can enhance the success of treatment outcomes in these deceptively complex teeth.

## Introduction

The mandibular premolars are among the most challenging teeth in endodontics due to their unpredictable root canal morphology. Although they are often considered straightforward because of their typically single-rooted anatomy, numerous studies have demonstrated wide variations in root and canal configurations that can complicate both diagnosis and treatment. A thorough understanding of these patterns and their deviations is essential, as failure to identify an additional canal may compromise treatment outcomes [1,2].

Large epidemiological studies confirm the clinical importance of this complexity. An analysis from the University of Washington reported mandibular first premolars to have the highest nonsurgical root canal treatment failure rate (11.45%), a finding attributed to the frequent difficulty in detecting lingual canals [2]. Similarly, multiple investigations have highlighted the morphological diversity of mandibular first premolars. Alfawaz et al. (2019) found that while a single root was present in 97.5% of cases, 1.5% exhibited two roots and 1% exhibited three roots [3]. In terms of canal number, Algarni et al. (2021) reported that 68% contained a single canal, 23.1% had two canals, and 8.3% presented with three canals [4].

From a clinical standpoint, identifying aberrant anatomy is often difficult because conventional periapical radiographs offer limited two-dimensional visualization, and symptoms may mimic those of more straightforward canal systems. This complexity increases the risk of iatrogenic errors such as ledging, perforation, over-instrumentation, or inadequate disinfection. Inadequate understanding of potential canal configurations can therefore compromise both the biomechanical preparation and the long-term prognosis of the tooth. Recent advances, such as cone-beam computed tomography (CBCT), magnification, and enhanced illumination, now enable clinicians to detect these variations more reliably, but their routine use must be guided by a strong clinical suspicion, which requires awareness of how commonly and how subtly these variations occur.

The mandibular second premolars also demonstrate variability, with studies suggesting differences influenced by both gender and ethnicity. These variations occur more frequently in males and in individuals of African descent, further underlining the importance of population-specific anatomical awareness [5]. Collectively, these studies establish that while the majority of mandibular premolars conform to the expected single-canal pattern, clinically significant proportions present with complex anatomy that can challenge even experienced practitioners.

The present case series aims to illustrate the successful nonsurgical management of three mandibular premolars with varied anatomy. It underscores the role of CBCT, enhanced magnification, and bioceramic obturation techniques in addressing such anatomical challenges and achieving predictable clinical outcomes.

## Case presentation

Case series

The cases presented in this series were selected from routine clinical work carried out in the Department of Conservative Dentistry and Endodontics. Selection was based on the presence of atypical clinical or radiographic features that raised suspicion of complex root canal anatomy, such as unusual root outlines, non-standard canal configurations, or persistent symptoms despite previous treatment. Only cases where patients provided informed written consent for both treatment and publication were included.

Case 1

A 44-year-old female patient reported to the Department of Conservative Dentistry and Endodontics with the chief complaint of a cavitated tooth in the left lower back region, with a history of pain radiating to the ear. The patient was undergoing fixed orthodontic treatment from a private clinic.

**Figure 1:**
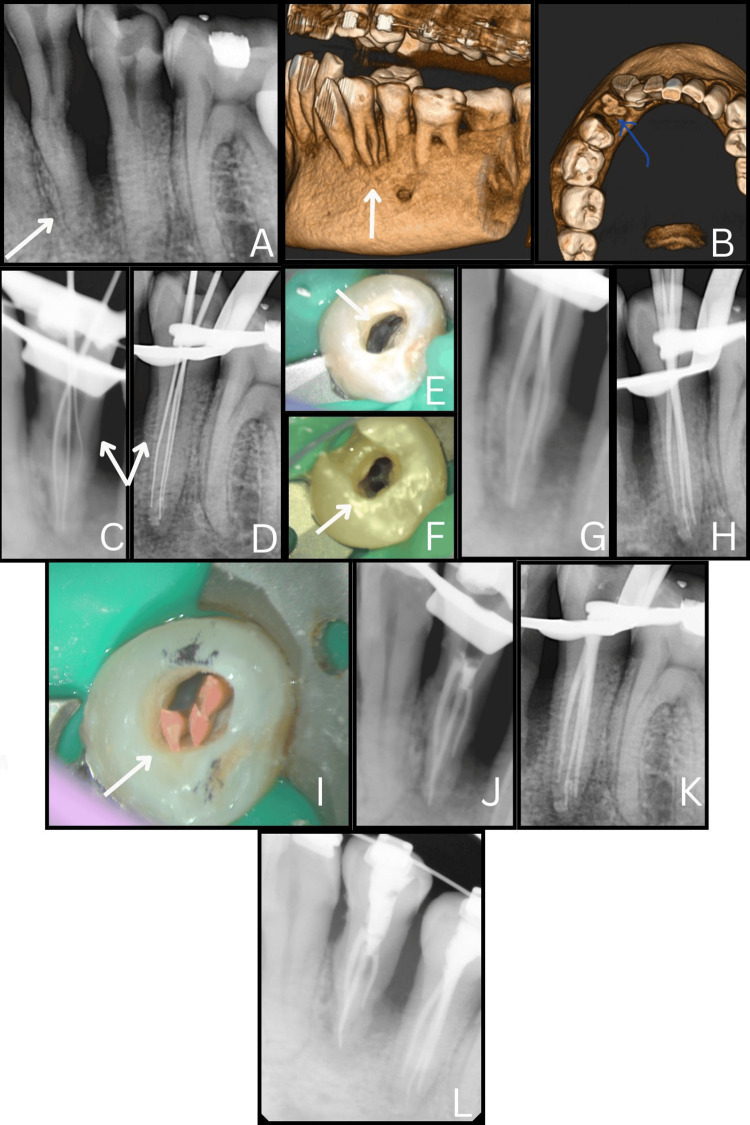
Diagnosis and management of teeth #34 and #35 (a) Preoperative periapical radiograph of teeth #34 and #35 showing altered root outlines suggestive of canal variation (arrow). (b) CBCT 3D reconstruction and axial sections confirming the presence of three distinct canals within two separate roots (arrows). (c, d) Working length radiographs of #34 and #35 respectively, illustrating negotiated canal pathways (arrows). (e, f) Clinical images under 2.5× magnification demonstrating refined access and canal orifice identification in #34 and #35 (arrows). (g, h) Master cone radiographs for #34 and #35 confirming canal patency and obturation length. (i) Clinical confirmation of master cone placement in #34. (j, k) Post-obturation radiographs of #34 and #35 demonstrating three-dimensional obturation quality. (l) Final radiograph showing completed post-endodontic restorations of both teeth.

Clinical examination revealed occluso-proximal caries related to teeth #34 and #35. Pulp sensitivity cold test of the teeth showed exaggerated response, with pain on percussion. During the first visit, a preoperative periapical radiograph was taken, which showed a long and deep pulp chamber and the presence of two separate root outlines in both teeth (Figure 1A). Eventually, teeth #34 and #35 were diagnosed with necrotic pulp with symptomatic apical periodontitis. A pre-operative CBCT scan was done to rule out the number of roots and canals. The 3D reconstruction and scan sections revealed the presence of two roots and three canals (one buccal and two lingual) in both teeth #34 and #35 (Figure 1B). CBCT was used selectively to minimize radiation exposure and was performed only in Case 1, where conventional radiographs could not clearly define root morphology. In the remaining cases, canal anatomy was adequately identified under the dental operating microscope (DOM).

Following a comprehensive assessment of the clinical condition, a nonsurgical root canal procedure was initiated. Local anaesthesia was administered using an inferior alveolar nerve block with 2% lidocaine containing 1:100,000 epinephrine. The involved teeth (#34 and #35) were isolated using a rubber dam (Coltene Whaledent Inc., Germany), and an access cavity was prepared to reach the pulp chamber. In #34, 2 canal orifices were readily visible under magnification ×2.5, while the third was located with the help of a dental operating microscope (Labomed Dental Microscope Prima DNT, USA) (Figure 1E). Similarly, two separate orifices were also located in #35 under magnification (Figure 1F). The working length of the canals in #34 and #35 was established using an electronic apex locator (Root ZX, J. Morita Corp, Tokyo, Japan), and further verified by a radiograph taken with a 10K-file (Mani, Prime Dental Product Pvt. Ltd., India) in place (Figures 1C, D).

Chemo-mechanical preparation of the root canal was done with JIZAI rotary files (Mani, Tochigi, Japan). In addition, 2.5% sodium hypochlorite (Prime Dental, India), 3 mm above the working length using a 30G double‑side vented needle (SuperEndo, India), followed by intracanal heating of sodium hypochlorite using a heated plugger (Fi‑P Heating and Packing Instrument, Woodpecker Medical Instrument Co, Guilin, China) 3 mm from the working length and activation 3 mm above the working length in each canal and premixed calcium hydroxide dressing (NeoCal, Orikam, India) was given for 1 week.

During the second visit, calcium hydroxide dressing was washed out from the canal using saline, followed by 17% ethylenediaminetetraacetic acid (NeoEDTA, Orikam, India) for the elimination of the smear layer, and at the end, rinsed with sterile saline. The canal was dried with paper points (Diadent, Chungju, Korea), and a master cone radiograph was taken (Figures 1G-1I). The canals were obturated with BioCeram Sealer (BioActive RCS, SafeEndo, India) and BioCeram Gutta-Percha points (EndoSequence BC Points, Brasseler, USA) (Figures 1J, 1K). The access was immediately sealed with bonded composite (Tetric N‑Ceram, Ivoclar Vivadent, Schaan, Liechtenstein) (Figure 1L), and the patient remained asymptomatic with no clinical symptoms and complications during the six-month follow-up period.

Case 2

**Figure 2:**
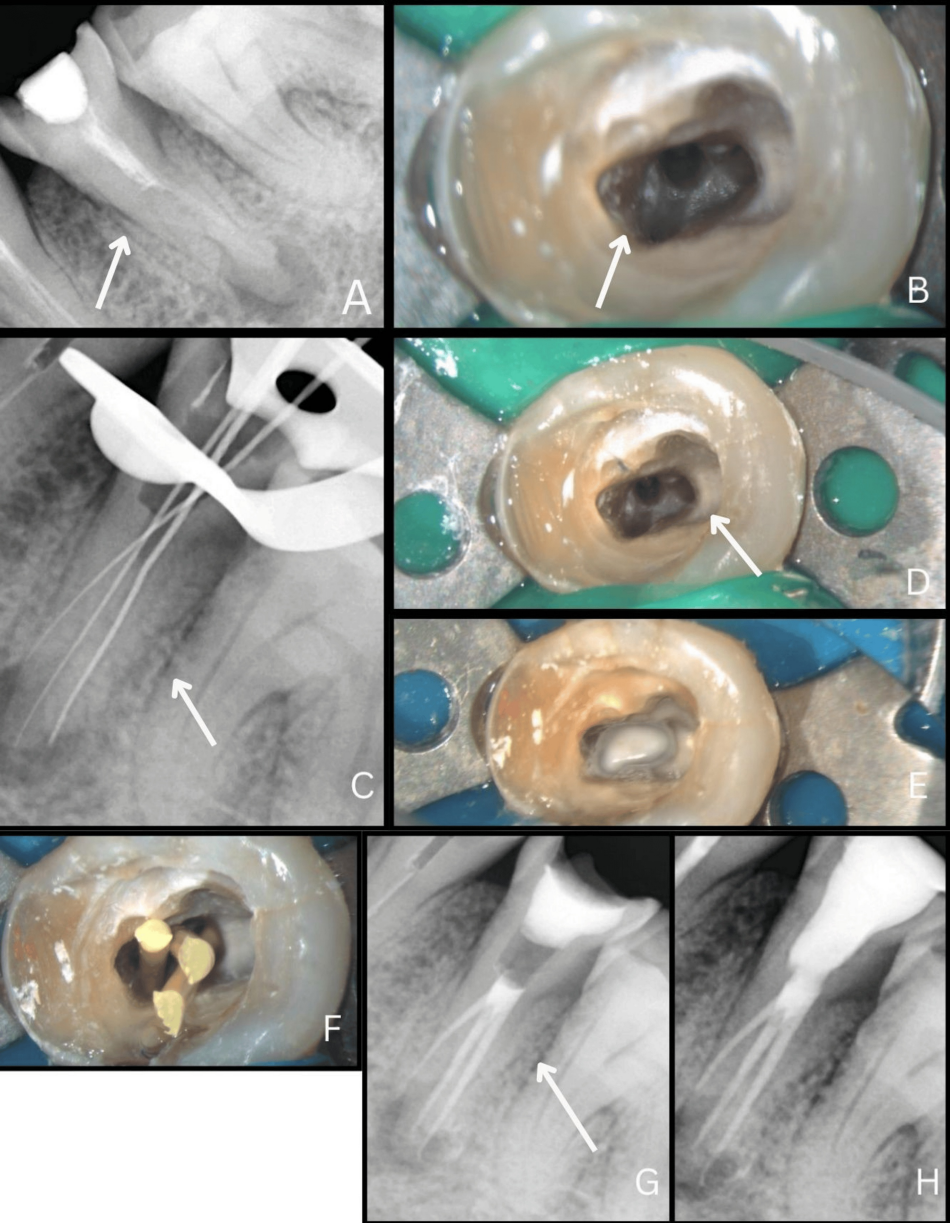
Diagnosis and management of tooth #35 with aberrant anatomy (a) Preoperative periapical radiograph of tooth #35 showing an atypical root outline suggestive of canal variation. (b) Clinical image under 2.5× magnification demonstrating initial access and identification of canal orifices. (c) Working length radiograph confirming the presence of three distinct canals. (d) Canal orifice configuration following biomechanical preparation. (e) Magnified clinical image showing placement of intracanal medicament within all canals. (f) Master cones positioned to verify canal shaping and working length. (g) Post-obturation radiograph demonstrating three-dimensional filling of all canals. (h) Final radiograph following composite restoration of #35.

A 33-year-old male patient was referred to the Department of Endodontics with the chief complaint of pain in the lower left back teeth and a history of previously initiated root canal treatment. Clinical examination revealed caries in tooth #35 with an access opening previously done and sealed with a temporary restoration. Radiographic examination showed periapical radiolucency in #35, the presence of radiopacity in the canal suggestive of an intracanal medicament, and more than one root canal was suspected (Figure 2A).

A pulpal diagnosis of symptomatic irreversible pulpitis with apical periodontitis was established, and nonsurgical endodontic treatment was planned for #35. After the administration of the local anesthetic agent (2% lidocaine with 1:100,000 epinephrine), the access preparation for tooth #35 was modified under a dental operating microscope (Labomed Dental Microscope Prima DNT). Three distinct canals were located at x2.5 magnification (Figure 2B) and irrigated with normal saline.

Working length was established with the use of an apex locator (Root ZX Mini, J. Morita Inc., USA) and confirmed by a radiograph (Figure 2C). The canals of tooth #35 were cleaned and shaped with hand K-files (Mani, Prime Dental Product Pvt. Ltd., India) and Mani Jizai (Mani, Tochigi, Japan) till size 25/0.04 and visualized under magnification (Figure 2D). The canals were irrigated with 2.5% sodium hypochlorite during instrumentation (Prime Dental, India), using a 30G double‑side vented needle (SuperEndo, India), followed by intracanal heating of sodium hypochlorite using a heated plugger (Fi‑P Heating and Packing Instrument, Woodpecker Medical Instrument Co) 3 mm from the working length along with ultrasonic activation (COXO, India) and premixed calcium hydroxide dressing (NeoCal, Orikam, India) was given for one week (Figure 2E).

On the recall appointment, calcium hydroxide was washed out from the canal using saline, followed by 17% ethylenediaminetetraacetic acid (NeoEDTA, Orikam), followed by sterile saline. The canal was dried with paper points (Diadent), and a master cone radiograph was taken (Figure 2F). The canals were obturated with BioCeram Sealer (BioActive RCS) and BioCeram Gutta-Percha points (EndoSequence BC Points), and a post-obturation radiograph was taken (Figure 2G). The access was restored with bonded composite resin (Tetric N‑Ceram) (Figure 2H). The patient was recalled after six months and was completely asymptomatic.

Case 3

A 27-year-old female presented to the Department of Endodontics with complaints of pain in the lower left posterior jaw region persisting for the past 10 days. On clinical examination, a deep mesio-proximal carious lesion was observed in tooth #34. Her medical and family history were non-contributory to the dental condition. Tenderness on percussion was noted in the affected tooth, indicating the presence of periodontal involvement. A pulp vitality test elicited an intense, painful response. Radiographic assessment revealed the presence of pulpal involvement along with peri-radicular radiolucency in #34. Based on the clinical and diagnostic findings, a diagnosis of symptomatic irreversible pulpitis accompanied by apical periodontitis was established.

**Figure 3:**
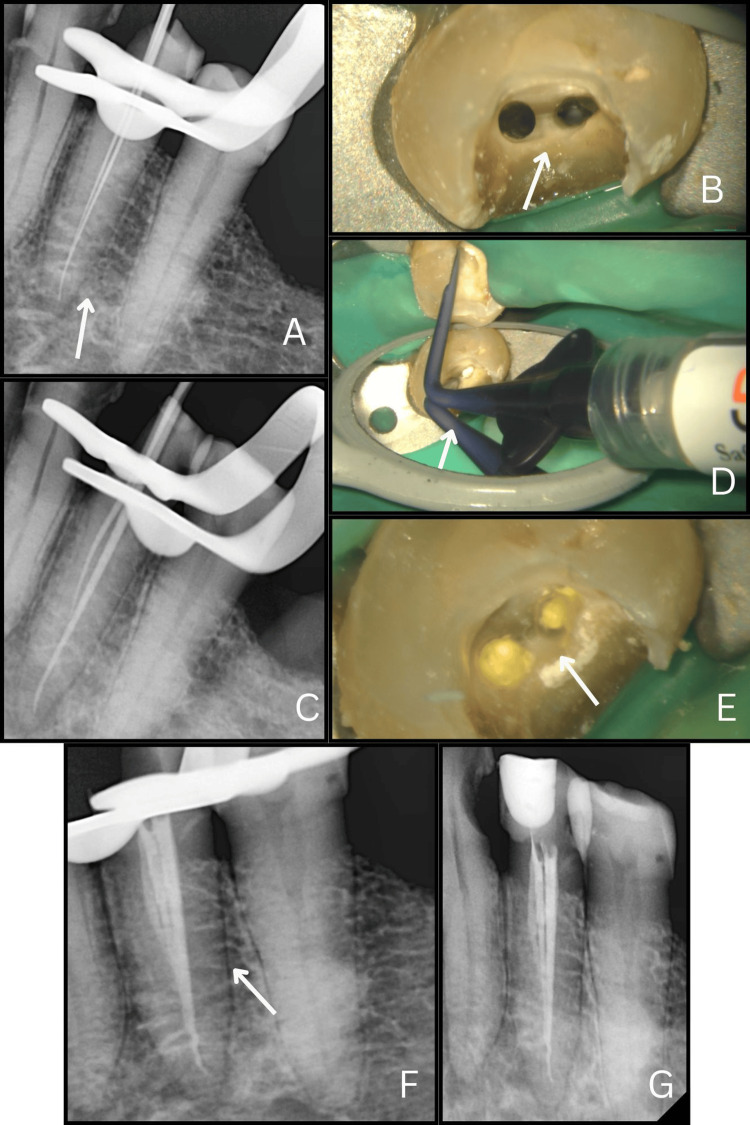
Magnification-guided diagnosis and management of tooth #34 (a) Working length radiograph of tooth #34 demonstrating a 2:1 canal configuration. (b) Clinical image under 2.5× magnification showing refined access and canal orifice identification. (c) Master cone radiograph confirming canal shaping and working length. (d) Magnified clinical image illustrating the injection of bioceramic sealer into the prepared canals. (e) Post-obturation clinical view showing canal filling and coronal sealing. (f) Post-obturation radiograph demonstrating complete three-dimensional obturation of #34. (g) Final radiograph following composite restoration of the tooth.

The patient was advised to undergo non-surgical root canal therapy, and informed consent was obtained prior to beginning the procedure. Profound anesthesia was administered using 2% lignocaine hydrochloride with adrenaline at a concentration of 1: 100,000. The procedure was performed under rubber dam isolation (Coltene Whaledent Inc., Germany). Carious tissue was entirely removed, and an access cavity was prepared under a dental operating microscope (Labomed Dental Microscope Prima DNT), during which two separate canals were identified in tooth #34. Canal patency was verified with a #10 K-file (Mani Inc., Tochigi, Japan), and initial working lengths were established using a #15 K-file along with Root ZX Mini Apex Locator (J. Morita Corp.), and later confirmed radiographically (Figure 3A). The canal morphology corresponded to Vertucci’s Type II configuration and was visualized at x2.5 magnification (Figure 3B) [6]. Biomechanical preparation was carried out using both manual K-files and nickel-titanium rotary instruments (Jizai, Mani Inc.), accompanied by copious irrigation with 2.5% sodium hypochlorite, 17% EDTA, and saline using a 30-gauge side-vented needle (SuperEndo, India). Sodium hypochlorite was heated within the canal (Fi‑P Heating and Packing Instrument, Woodpecker Medical Instrument Co) along with ultrasonic activation 3 mm short of the apex. The master cone fit was confirmed radiographically at the final working length (Figure 3C). After drying the canals with sterile paper points, obturation was done using bioceramic sealer (BioActive RCS, SafeEndo) (Figures 3D-3F), followed by composite restoration (Tetric N Ceram, Ivoclar Vivadent). A final radiograph, including a horizontal view, confirmed a hermetic seal (Figure 3G).

## Discussion

Successful root canal therapy relies on the precise location, cleaning, shaping, and sealing of all root canals. A failure in any of these essential steps can result in persistent symptoms, post-treatment disease, or complications involving the treated tooth [7]. Effective treatment of intricate root canal anatomies requires a high level of clinical proficiency combined with the effective use of advanced endodontic methods.

The literature exhibits a kaleidoscope of variations in the lower bicuspids. This vast variation is responsible for the high failure rate. Also, in these teeth, root and canal divisions typically take place in the middle or apical third of the root, making it challenging to identify such anatomical variations [8].

In Case 1, the first and second left mandibular premolars have two roots with three root canals, as seen on CBCT. This type of morphology is similar to the reported case in Poorni S et al. [9]. On the other hand, one recent case in China reported the same morphology in mandibular first premolars with two roots and three root canals [10].

In Case 2, the second mandibular left premolar was also two-rooted with three root canals, which is similar to the case by Dua R et al., who reported that double-rooted mandibular premolars have an incidence of 1.8% [11].

In Case 3, the canal configuration followed a 2: 1 pattern with double canals merging into one. Such anatomical variation has an incidence of up to 9% [12]. It has been demonstrated in literature multiple times, as in the case reports by Mittal N et al. (2020) [13].

Reports of similar morphologies in mandibular premolars, such as those described by Poorni et al., Dua et al., and Mittal et al., confirm that these anatomical variations, while uncommon, are clinically significant [9,11,13]. Teeth with additional roots or canals require prolonged chairside time, as canal location and negotiation are considerably more difficult than in standard anatomy. The increased complexity also elevates the risk of procedural complications, including instrument separation in narrow or curved canals, ledge or perforation formation during instrumentation, and missed canals, leading to persistent periapical disease. Such risks highlight the importance of thorough preoperative assessment, careful canal exploration under magnification, and adjunctive imaging when indicated. By anticipating these challenges, clinicians can allocate appropriate treatment time and adopt strategies that minimize complications, thereby improving long-term prognosis.

The three reported cases involving mandibular premolars revealed distinct configurations of apical canal terminations. In the first scenario, the tooth exhibited a full trifurcation at the mid-root level, with each root harboring its own canal. The second case showed a dual-rooted premolar with three separate canals, each terminating independently at the apex. In contrast, the third case involved a single-rooted premolar containing two canals within the same root structure. Such anatomical diversity, especially regarding root form, apical third complexities, and individual canal exits, can greatly impact the outcome and longevity of root canal therapy.

Accurate interpretation of conventional periapical radiographs taken in more than one angle is extremely essential to detect any morphological variations of teeth [14]. When standard radiographic methods do not yield clear or detailed insights, CBCT serves as an essential tool for uncovering complex root canal anatomies. CBCT was taken in the first case due to difficulty in identifying canals [15]. The cases reported were all visualized under the dental operating microscope. The use of a dental operating microscope significantly enhances the precision of canal location during endodontic procedures. Its magnification and illumination allow for a clearer view of the pulpal floor, helping clinicians identify additional or aberrant canal orifices that may be missed with the naked eye [16].

The canals were prepared upto size 25/0.04 using the Jizai, Mani rotary system, followed by copious irrigation with sodium hypochlorite, which was activated using an ultrasonic system for thorough disinfection. The combination of sodium hypochlorite and ultrasonic activation produces a synergistic effect, resulting in enhanced canal disinfection, more effective elimination of pulp tissue and dentinal debris, and improved smear layer removal [17].

The technique used in all cases was single cone obturation, as it is less technique-sensitive, much easier, and less time-consuming. The application of Bioceramic sealers functions as a bonding interface between the gutta-percha cone and the canal wall. They infiltrate deeply into the dentinal tubules, forming a robust mechanical interlock that reinforces the attachment of the filling material to the canal structure [18]. Although the single-cone technique with bioceramic sealers is simple and efficient, its main limitation in complex anatomies is the risk of voids and incomplete adaptation to canal walls. In contrast, warm vertical compaction provides superior filling of irregularities and a more homogenous seal, but it is technique-sensitive, requires specialized equipment, and carries a higher risk of extrusion. Thus, single-cone obturation remains practical for routine cases, while warm vertical compaction may be preferred in teeth with highly irregular canal systems [19].

The main limitation of this case series is the small sample size, which restricts the applicability of the findings to broader populations. Another limitation is the selective use of CBCT and magnification, which were not consistently applied in all cases.

## Conclusions

This case series demonstrates that mandibular premolars may present with unexpected canal configurations that challenge routine diagnosis and treatment. While CBCT and the dental operating microscope proved useful in clarifying anatomy when conventional assessment was inconclusive, their effectiveness in this report should be interpreted as case-specific observations rather than generalizable evidence. Similarly, the obturation techniques and materials employed reflect individualized clinical decisions rather than comparative superiority. Given the small sample size and limited follow-up data, no assumptions about treatment predictability can be made. These cases underscore the importance of careful radiographic interpretation, meticulous tactile examination, and tailoring treatment strategies to the specific anatomy encountered, while highlighting the need for larger, standardized studies to better define best practices for managing complex mandibular premolar anatomies.
